# RNA sequencing analysis of human podocytes reveals glucocorticoid regulated gene networks targeting non-immune pathways

**DOI:** 10.1038/srep35671

**Published:** 2016-10-24

**Authors:** Lulu Jiang, Charles C. T. Hindmarch, Mark Rogers, Colin Campbell, Christy Waterfall, Jane Coghill, Peter W. Mathieson, Gavin I. Welsh

**Affiliations:** 1 Bristol Renal, School of Clinical Sciences, University of Bristol, Bristol, UK; 2 Department of Biomedical and Molecular Sciences, Queen’s University, Kingston, ON, Canada; 3 Department of Physiology, Faculty of Medicine, University of Malaya, Kuala Lumpur, Malaysia; 4 Department of Engineering Mathematics, University of Bristol, Bristol, UK; 5 Bristol Genomics Facility, School of Biological Sciences, University of Bristol, Bristol, UK; 6 President’s Office, The University of Hong Kong, Hong Kong, China

## Abstract

Glucocorticoids are steroids that reduce inflammation and are used as immunosuppressive drugs for many diseases. They are also the mainstay for the treatment of minimal change nephropathy (MCN), which is characterised by an absence of inflammation. Their mechanisms of action remain elusive. Evidence suggests that immunomodulatory drugs can directly act on glomerular epithelial cells or ‘podocytes’, the cell type which is the main target of injury in MCN. To understand the nature of glucocorticoid effects on non-immune cell functions, we generated RNA sequencing data from human podocyte cell lines and identified the genes that are significantly regulated in dexamethasone-treated podocytes compared to vehicle-treated cells. The upregulated genes are of functional relevance to cytoskeleton-related processes, whereas the downregulated genes mostly encode pro-inflammatory cytokines and growth factors. We observed a tendency for dexamethasone-upregulated genes to be downregulated in MCN patients. Integrative analysis revealed gene networks composed of critical signaling pathways that are likely targeted by dexamethasone in podocytes.

Glucocorticoids (GCs) are a class of steroid hormones used as frontline immunomodulatory drugs in the treatment of many inflammatory diseases owing to their potent effects on immune cells. However, GCs are also therapeutically effective in Minimal Change Nephropathy (MCN) which is characterised by a lack of inflammation in the kidney. This raises the question as to how GCs exert their therapeutic efficacy on non-immune cells such as those in the kidney.

MCN is the most common cause of nephrotic syndrome in children, accounting for approximately 80% of cases. Patients with MCN suffer from massive leakage of protein from the blood stream into the urine, resulting from dysfunction of the glomerular filtration barrier (GFB) of the kidney. The GFB functions as a blood filter, preventing protein loss into urine (Fig. 1A). It is mainly composed of two cell types: glomerular endothelial cells and glomerular epithelial cells (or ‘podocytes’), separated by a specialized glomerular basement membrane (GBM).

Increasing evidence points to the importance of podocytes in glomerular disease. Mutations in podocyte-specific genes^1^,^2^,^3^,^4^ cause congenital or early-onset nephrotic syndrome. Proteins encoded by many of these genes reside in the slit diaphragm - a structure connecting adjacent podocyte foot processes. They interact with other proteins to regulate the podocyte actin cytoskeleton which underlies the function of these cells and the glomerulus^5^,^6^. In addition to their importance in glomerular function, we and others have also shown that podocytes are a promising cellular target for immunomodulatory drugs in the treatment of glomerular diseases. In 2008, PW Mathieson^7^ proposed that drug therapies for glomerular diseases may exert some or all of their therapeutic effects via direct actions on the glomerular podocytes rather than via the modulation of immune cell function. Since then numerous studies have demonstrated that these drugs do act directly on podocytes^8^,^9^,^10^, and that their efficacy in treating proteinuria can be independent of their effects on immune cells^11^.

The underlying molecular mechanisms as to how GCs exert their therapeutic effects on podocytes are still unclear. To address this, we utilised our established conditionally immortalized human podocyte cell lines for RNA sequencing (RNA-seq) to generate a genome-wide expression profile of the effects of dexamethasone on human podocytes derived from three different healthy kidney donors. We further demonstrate the usefulness of this resource in revealing transcriptome features that may explain the efficacy of glucocorticoids in disease. In a wider context, our results advance the knowledge of glucocorticoids targeting non-immune cell functions.

## Results and Discussion

### RNA sequencing analysis of podocytes treated with dexamethasone

**Figure 1:**
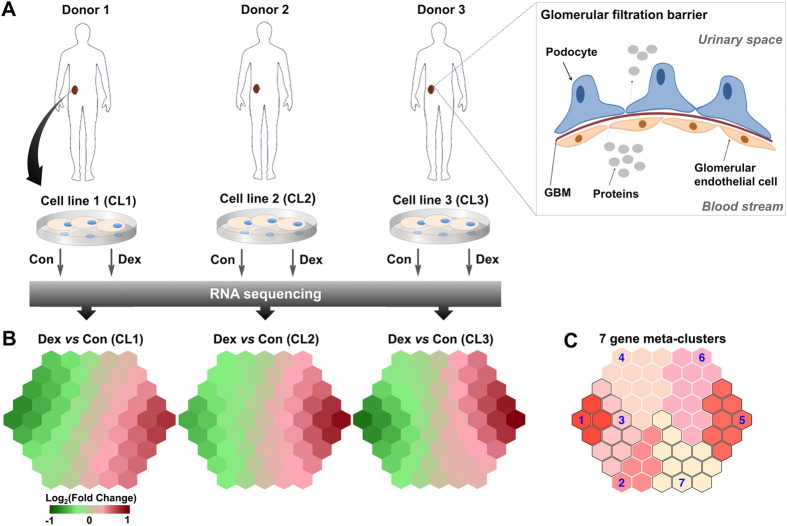
RNA sequencing analysis of 3 podocyte cell lines derived from independent healthy donors. (**A**) Experimental design. Conditionally immortalized human podocytes were developed from 3 kidney transplant donors. Differentiated cells were treated with PBS or 0.1 μM dexamethasone for 24 hours, which were then subjected to RNA extraction and sequencing. On the top right illustrates the structural composition of glomerular filtration barrier and the occurrence of protein loss into urine in disease. Notation: Con, control; Dex, dexamethasone; GBM, glomerular basement membrane; CL1-3, cell lines 1–3. (**B**) Cell line-specific transcriptome changes in response to dexamethasone. Clustering and visualization of differentially expressed genes were analysed using a supra-hexagonal map. Genes with similar patterns across cell lines are mapped onto the same or nearby regions in the map. (**C**) A map illustrating 7 gene meta-clusters.

We have used 3 human podocyte cell lines for sequencing which were derived from the kidneys of 3 independent healthy donors. Based on three biological replicates in a paired design (Fig. 1A), we identified 2,276 genes in podocytes that were significantly regulated upon dexamethasone treatment. These genes were then analysed using a supra-hexagonal map for gene clustering and visualization. Cell line-specific transcriptome changes are illustrated in Fig. 1B, in which genes with similar patterns across cell lines are mapped onto the same regions in the map. Comparison of these illustrations demonstrates a considerable consistency of expression changes across the cell lines. To better reveal inherent relations between these genes, we applied a topology-preserving clustering procedure identifying seven gene meta-clusters (Fig. 1C): highly downregulated (cluster 1), downregulated (clusters 2–4), highly upregulated (cluster 5), and upregulated (clusters 6–7). Genes within each of the seven clusters displayed highly similar expression patterns in all cell lines, suggesting that they share common features. Detailed information about these genes and their cluster memberships can be found in Supplementary Table 1.

### Enrichment analysis reveals functional characteristics of dexamethasone–treated podocytes

**Figure 2:**
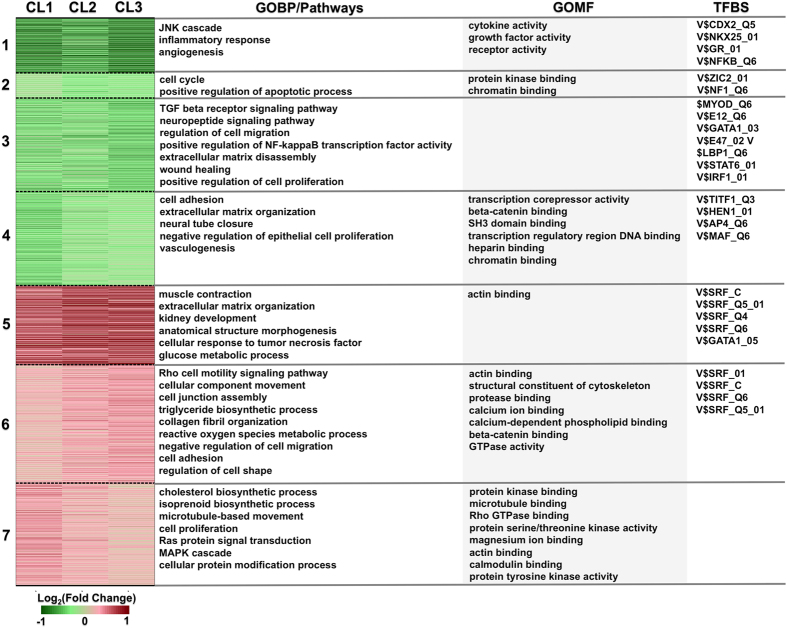
Enrichment analysis of the clustered genes. Functional enrichment analysis for genes in each of the 7 clusters is based on Gene Ontology molecular function (GOMF) and biological process (GOBP). KEGG, BioCarta and Reactome pathways are used for pathway enrichment analysis, and transcription factor binding sites (TFBSs) for TF enrichment analysis. Notation: CL1–3, cell lines 1–3.

To reveal the functional relevance of dexamethasone-regulated genes in podocytes, we next performed enrichment analysis of genes in each of the 7 clusters using Gene Ontology (GO), BioCarta, KEGG and Reactome pathways, and transcription factor binding sites (TFBSs). The GO terms, pathways and TFBSs that were significantly associated with the gene clusters are illustrated in Fig. 2, and the functional characteristics identified are summarized below.

#### Dexamethasone-downregulated genes are largely pro-inflammatory cytokines and growth factors

Intriguingly, we found that genes repressed in cluster 1–4 by dexamethasone included a wide variety of genes encoding pro-inflammatory cytokines including chemokines (*CCL2, CXCL12*), interleukins (*IL11, IL6, IL23A*), *TNF, TGFB1, CSF2*, and some interferon regulatory factors (*IRF2, IRF6*). Fibroblast growth factors (*FGF2, FGF5*) and vascular endothelial growth factors (*VEGFA, VEGFC*) were also suppressed. We have previously reported that dexamethasone downregulates VEGF in podocytes^12^, thus here VEGF genes serve as an internal control for our sequencing data. Furthermore, as shown in Fig. 2, the GO pathways observed in cluster 1–4 included the JNK cascade and inflammatory responses that are the classic pathways by which GCs universally exert suppressive effects. The transcription factors enriched from the TFBSs analysis are diverse and include NFκB and AP1 (Supplementary Table 2), crucial transcriptional regulators of pro-inflammatory genes, which are known to be the transrepression targets of the glucocorticoid receptor (GR) for its anti-inflammatory effects^13^. Interestingly, podocytes appear to be capable of expressing and producing cytokines such as *IL-1* and *CXCL12*^14^,^15^ and upregulating the NFκB cascade signaling during injury^16^ which could consequently contribute to glomerular injury^17^. In this case, repression of cytokine genes and related pathways in podocytes might represent part of the mechanism by which glucocorticoids exert benefits in certain glomerular diseases such as MCN.

#### Dexamethasone-upregulated genes are linked to cytoskeleton-related process

Genes in cluster 5–7 are related to the structure of the podocyte. A large number of these genes encode actin binding proteins (*MYO1C, MYO9A*), structural constituents of the cytoskeleton (*MSN, TUBB, TUBA1B*), and actin regulatory proteins (*INF2, ROCK1, PPP1CB, PPP2CB*) (Table 1). Podocytes contain microtubules and intermediate filaments in their cell bodies and major processes for structural support for the cell, and microfilaments in the foot processes forming the highly delicate cytoskeletal architecture that underlies the function of the cell. A common pathological feature of nephrotic syndrome is podocyte foot process effacement. When the cytoskeleton network in podocytes is disrupted, the cells become flat and more motile, leading to proteinuria. Thus, maintaining the podocyte cytoskeleton is crucial for normal functioning glomerular filters^18^. Interestingly, both microtubule (α-and β-tubulin) and microfilament (α-actinin and myosin) encoding genes were found to be upregulated by dexamethasone (Table 1). In addition, *KIF23*, encoding CHO1/MKLP1, a kinesin-like protein that is essential for process formation^19^, and *MAP4*, encoding microtubule-associated protein which promotes microtubule assembly and stabilization^20^ were also induced. *PPP2CB* encodes phosphatase 2A (PP2A) catalytic subunit. Interestingly, in podocyte expression of this protein appears to be developmentally regulated^21^: *in vivo*, only process-forming cells express high level of PP2A^22^,^23^. Inhibition of PP2A in cultured podocytes abolishes process formation and causes abnormal microtubule assembly^24^. Therefore, upregulation of PP2A by dexamethasone could promote podocyte process formation and potentially attenuate podocyte injury during disease. *EZR* (encoding protein Ezrin) is another important gene induced by dexamethasone. Ezrin is an actin-binding protein that connects plasma membrane and actin cytoskeleton as an intermediator, and has been reported to be downregulated in diabetic glomeruli^25^. Furthermore, the transcription factor SRF (serum response factor) was found to be significantly enriched in cluster 5 and 6. SRF is reported to be a master regulator of actin cytoskeleton^26^ which can be activated by RhoA during filamentous actin (F-actin) polymerization^27^,^28^. A low level of RhoA causes the loss of podocyte stress fibers and is associated with foot process effacement^29^ which is a typical feature of MCN^30^. A large number of SRF-targeted genes were upregulated by dexamethasone, implicating the important role it may play in the actin regulation in podocytes. Our observations on the dramatic effects of dexamethasone on podocyte actin related genes are consistent with previous studies^31^,^32^,^33^ showing that dexamethasone protects and stabilizes podocyte actin cytoskeleton. Taken together, our data suggest that effects of dexamethasone on the podocyte cytoskeleton may involve the regulation of both microtubules and actin filaments, which could maximally promote the stability of the cell structure and function.

#### Dexamethasone regulates genes that are involved in podocyte differentiation

Of note, dexamethasone has been reported to restore the podocyte differentiation markers and ameliorate podocyte injury in proteinuric murine models^34^. Podocyte-specific transcription factor Wilm’s Tumor protein 1 (WT1) plays crucial role in glomerular differentiation and podocyte function^35^,^36^. At the early stage of podocyte differentiation, the expression of WT1 is highest^37^. AREG (amphiregulin) is a known target gene of WT1 and it enhances branching morphogenesis of ureteric bud during kidney differentiation^38^,^39^. Of interest, we noted that AREG was significantly upregulated by dexamethasone (Supplementary Table 1). Similarly, there was also an upregulation in FOXC2 (Supplementary Table 1), a transcription factor known to be involved in the podocyte specification^40^ and which cooperates with FOXC1 to maintain podocyte function^41^. Combined knockdown of WT1 and FOXC2 resulted in a loss of all podocyte marker gene expression^42^. This data may suggest the importance of these genes in promoting podocyte differentiation and cell function by dexamethasone.

### Comparative analysis identifies therapeutic candidate targets for MCN

**Figure 3:**
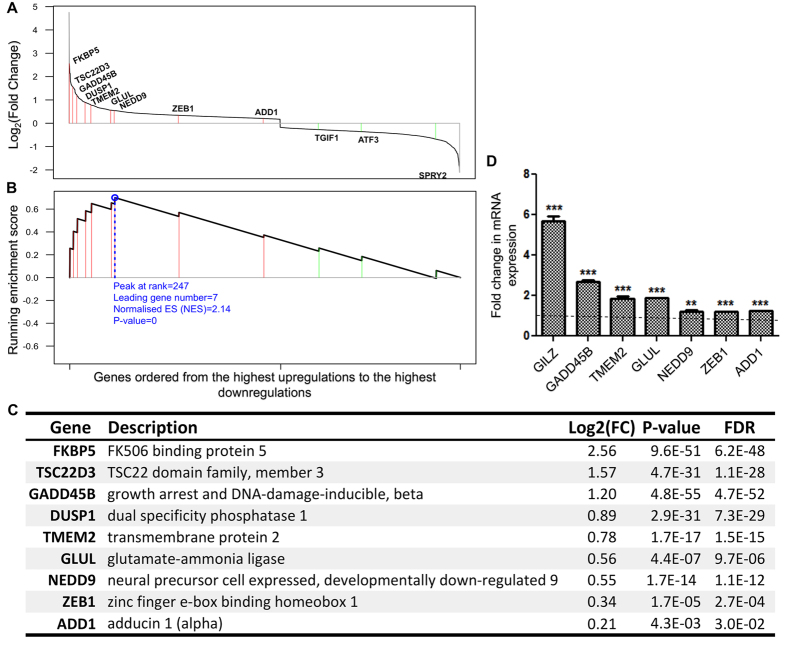
Comparative analysis of RNA-seq data with the transcriptome data of MCN patients. (**A,B**) GSEA shows that genes in the MCN-downregulated signature had a tendency of being significantly upregulated by dexamethasone, whereas no such tendency was observed for genes in the MCN-upregulated signature. (**C**) The list of 9 therapeutic candidate target genes identified as being repressed in MCN but upregulated by dexamethasone. (**D**) Real-time RT-PCR validation of the identified potential therapeutic targets. Cells were treated with vehicle or dexamethasone (0.1 μM) for 24 hours and then RNA was extracted and analyzed by real-time RT-PCR. Values were normalized to GAPDH and fold changes compared with vehicles were plotted as means ± SE from triplicates of four independent experiments. Dotted line indicates the basal expression. Notation: dexamethasone treatment versus vehicle (*); Significance: **P < 0.01, ***P < 0.001.

Dexamethasone-regulated genes represent a wide transcriptional spectrum characteristic of podocytes responding to glucocorticoids. To identify therapeutic candidate targets from this wide spectrum of genes, we further conducted a comparative analysis relative to the publically available transcriptome data of MCN patients. According to the Nephroseq database, we defined two MCN-specific gene signatures: a downregulated signature and an upregulated signature (Supplementary Table 3). Using GSEA we found that genes in the MCN-downregulated signature had a significant tendency of being upregulated by dexamethasone (Fig. 3A,B), whereas no such tendency was observed for genes in the MCN-upregulated signature. This implies that dexamethasone is able to reactivate gene expression programs that are suppressed in the disease state. Based on this finding, we identified 9 therapeutic candidate target genes that are repressed in MCN but are induced by dexamethasone (Fig. 3C).

#### Candidate genes that are associated with glucocorticoids’ immune-efficacy

Notably, among the 9 candidate genes, three genes (*FKBP5, TSC22D3* and *DUSP1*) are well-known glucocorticoid-induced genes that are involved in the immunosuppressive actions of glucocorticoids, but their roles in podocyte and glomerular function are unclear. FKBP5 (FK506 binding protein 51) is an hsp90 co-chaperone protein that is bound to GR and regulates GR sensitivity^43^. TSC22D3 (also known as GILZ, glucocorticoid-induced leucine zipper) inhibits the transcriptional activity of its target proteins such as NFκB^44^ and reduces the expression of costimulatory molecules CD80 and CD86 in macrophages and their production of chemokines^45^. In addition to mediating the anti-inflammatory effects, GILZ has also been reported to be involved in the regulation of T-helper cell differentiation^46^ and the increase of the epithelial Na^+^ transport in the kidney^47^. DUSP1 (dual specificity phosphatase 1) is a mitogen-activated protein kinase (MAPK) phosphatase that plays a partial role in dexamethasone-dependent repression of inflammatory genes^48^. DUSP1^−/−^ mice show high sensitivity to lipopolysaccharides (LPS) and exhibit significantly increased serum cytokine levels^48^. However the mechanisms by which these glucocorticoid-targeted genes play a role in the dysfunction of podocytes in MCN need to be established.

#### Candidate genes that are actin-related but novel in relation to podocytes

It is worth noting that 2 of the 9 candidate genes are actin-related genes (*NEDD9* and *ADD1*). NEDD9, also known as enhancer of filamentation 1 (EF1), localizes to^49^ and stabilizes focal adhesions and increases binding of the cell to extra cellular matrix (ECM)^50^. The absence of NEDD9 leads to an increased rate of focal adhesion disassembly and decreased adhesion to fibronectin in mouse embryo fibroblasts. β1 integrin activation is also significantly suppressed in NEDD9^−/−^ cells^50^. However the role of NEDD9 in regulating focal adhesion in podocytes has not been reported. ADD1 (Adducin 1) is an actin-binding protein that is important for stabilization of the membrane cortical cytoskeleton^51^,^52^ and cell-cell adhesion^53^,^54^. It was also reported to associate with mitotic spindles and interact with Myosin-X to regulate spindle assembly and mitotic progression^55^. Upregulation of *ADD1* and *NEDD9* in podocytes may suggest that dexamethasone is able to restabilize the actin skeleton by increasing the expression of actin components which are lost in disease, and may also strengthen the weakened binding to the ECM by mediating focal adhesions reestablishing the signaling transduction between the ECM and the actin cytoskeleton.

#### Candidate genes identified from transcriptome comparative analysis were further validated

Figure 3D shows the induced-expression of candidate genes by dexamethasone in podocytes validated by real-time RT-PCR (Upregulation of *FKBP5* and *DUSP1* has been reported in our previous study^8^ so they were excluded from validation here). In summary, these genes were identified to be repressed in MCN but activated by dexamethasone, suggesting that they play an important role in glomerular function, and may represent potential novel therapeutic targets.

### Integrative analysis identifies gene networks of dexamethasone targeting podocytes

**Figure 4:**
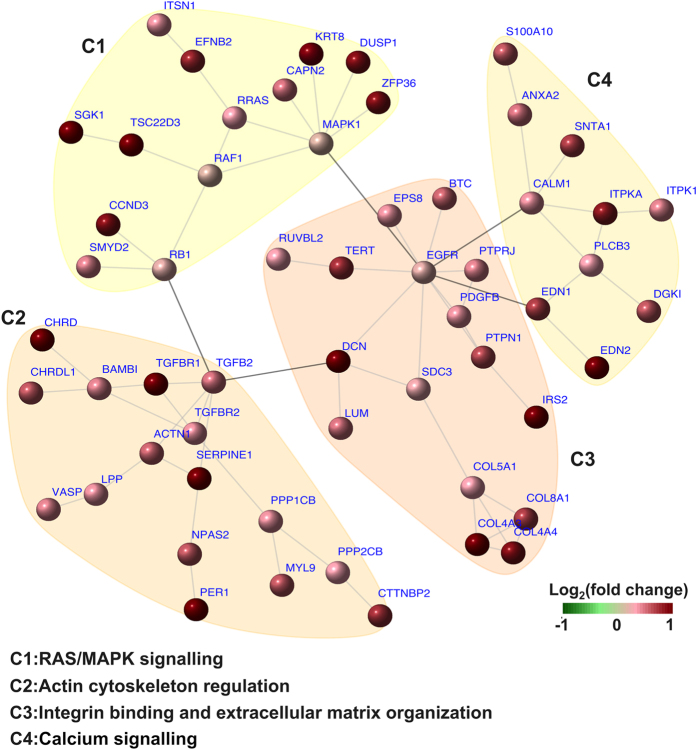
Gene network upregulated by dexamethasone in podocytes. The network is identified through integrative analysis of our RNA-seq expression data with known protein interaction data. Four communities were identified (C1–C4). Enrichment analysis shows the crosstalk of RAS/MAPK signalling, regulation of actin cytoskeleton and focal adhesion, and calcium signalling in this gene network.

It is likely that genes regulated by dexamethasone do not function alone but instead work together as a network. To identify such an interconnected-gene network we carried out integrative analysis through integration of our RNA-seq expression data with known protein interaction data. As a result, two networks were identified, one for downregulated genes and the other for upregulated genes. The downregulated-gene network signifies a broad-spectrum of effects of glucocorticoids on cell proliferation, survival, and immune-related process (see Supplementary Fig. 1 and Supplementary Table 4). The upregulated-gene network depicts a podocyte-specific interconnection between RAS/MAPK signaling, regulation of actin cytoskeleton and focal adhesion, and calcium signaling. These interactions are well captured by the community structure (communities C1–C4) (Fig. 4 and Table 2), demonstrating the key signaling network that dexamethasone positively regulates in podocytes.

#### Gene network negatively regulated by dexamethasone reveals the association of P53 signalling with cytokine and growth factor pathways in podocytes

The crosstalk between TP53 (P53) signaling pathway (C1), cytokine signaling (C2) and growth factor signaling (C3) were observed in the gene network downregulated by dexamethasone. The hub genes of respective communities mediate this crosstalk: *P53* in C1, *IL6* in C2 and *VEGFA* in C3. As shown in the Supplementary Fig. 1, C3 was tightly linked to C2 via direct interactions of *VEGFA* with multiple genes including *MMP2, IGFBP3, TNF, HMOX1, CXCL12* and *TGFB1*. GR activated by dexamethasone is reported to inhibit P53-induced cell cycle arrest and apoptosis by interacting with P53 and subsequently leading to inactivation of P53 via cytoplasmic sequestration^56^. The crosstalk between the P53 pathway and cytokine and growth factor signaling suggests that dexamethasone affects podocyte proliferation and survival by negatively regulating P53, and this negative regulation may also be associated with its repressive effects on cytokine and growth factor-related genes.

#### Gene network upregulated by dexamethasone illustrates podocyte-specific crosstalk of signaling pathways in response to glucocorticoid

As seen in Fig. 4, we found that the *EGFR* gene in the community of integrin binding and ECM organization (C3) directly connected to two other communities: the RAS/MAPK signaling pathway community (C1) and the calcium signaling community (C4), and indirectly linked to the actin regulation community (C2) via the *DCN* gene. The identification of *EGFR* as a hub gene is reasonable: integrin-mediated cell adhesion to ECM proteins induces partial activation of EGFR in epithelial cells, and this activation is required for multiple signal transduction events including the activation of Ras/Erk signaling^57^,^58^. *TGFB1* expression was suppressed by dexamethasone, however *TGGBR1, TGFBR2* and *TGFB2* were upregulated in C2, and *TGFB2* appeared to be a link for actin-related genes to connect to RAS/MAPK pathway via *RB1* and to integrin signaling pathway via the gene *DCN*. In addition, *EGFR* links *SDC3* (encoding syndecan 3, an adhesion receptor) and *SDC3* then connects to several collagen-encoding genes *COL5A1, COL4A3, COL4A4* and *COL8A1*. GBM (illustrated in Fig. 1A) is a dense network of ECM containing collagen IV and laminin and other structural and regulatory proteins^59^. The absence of the collagen IV α3α4α5 can cause Alport syndrome and progressive renal failure^60^,^61^. Interestingly dexamethasone upregulated *COL4A3* (collagen IV α3) and *COL4A4* (collagen IV α4) and also repressed the expression of some other collagen genes such as *COL6A3, COL14A1* and *COL27A1*. Notably, *EGFR* also links to C4 via *CALM1* (encoding calmodulin 1) indicating the important involvement of calcium signaling in the regulation of podocyte function by dexamethasone. Indeed, Ca^2+^ signaling is very important: the shape of each cell is regulated by Ca^2+^/calmodulin through controlling the interactions of myosin with cytoskeletal actin^62^,^63^.

Taken together, these two networks clearly illustrate the coordinated cellular response to dexamethasone treatment in podocytes. P53 may play an important role in mediating glucocorticoid-dependent effects on podocyte proliferation, survival and cytokine regulation. Integrin signaling exhibits as a central regulator that leads the crosstalk between RAS/MAPK signaling, regulation of actin, and calcium signaling, suggesting it is an important target for glucocorticoid action in podocytes. Importantly, *DUSP1* and *GILZ*, the genes repressed in disease, were found in the upregulated gene network, which raise the possibility of their functional connection with actin regulation and integrin signaling pathways.

## Conclusions

We are the first to apply RNA-seq in generating transcriptome data that is informative for understanding molecular mechanisms of GCs’ effectiveness in non-inflammatory glomerular disease. Our data illustrates that the effects of dexamethasone on podocytes in general function in two ways: 1) suppression of the cells’ capacity to produce pro-inflammatory mediators in response to injury to limit/prevent the damage to the glomerulus, and 2) enhancement of podocyte intrinsic function by promoting the stabilization of the actin cytoskeleton and its binding to GBM. In terms of MCN, the latter could be the main mechanism. These findings will advance our understanding of the efficacy of GCs in nephrotic syndrome. New candidate targets identified in this study will facilitate the development of novel podocyte-targeted treatment without causing systemic toxicity.

## Methods

### Podocyte culture and glucocorticoid treatment

All experimental protocols were approved by the NRES Committee South West-Central Bristol and were performed in accordance with Committee guidelines. Conditionally immortalized human podocytes were established from three kidney transplant donors with no kidney disease^64^. Cell lines were regularly tested and were mycoplasma-free. Cells were grown to 70–80% confluence at 33 °C in 5% CO_2_ before thermo-switching to 37 °C in 5% CO_2_ and allowed to differentiate for 10 days. Differentiated cells were then treated with PBS (vehicle) or 0.1μM dexamethasone (Sigma–Aldrich) for 24 hours, then subsequently used for RNA extraction and sequencing.

### RNA extraction, cDNA library construction and sequencing

Total RNA was extracted from control and dexamethasone-treated podocytes using the TRIzol method. RNA samples were then subjected to DNase treatment (Ambion TURBO DNase, Life Technologies) and purification (RNeasy Mini Kit, QIAGEN) according to the manufacturer’s instructions. The cDNA library preparation and the sequencing were performed following manufacturer’s instructions at the Bristol Genomics Facility, University of Bristol. Briefly, approximately 1μg of purified total RNA was prepared for sequencing using the Illumina TruSeq mRNA Seq v2 kit. Sequencing of 100bp paired-end reads was performed with the Illumina HiSeq 2500 instrument. The raw data was processed using Illumina software, RTA 1.17.21.3 with default filter and quality settings. The reads were demultiplexed (allowing no mismatches in the index sequence) with CASAVA 1.8.2.

### RNA-seq data mining

RNA-seq alignment and analysis was performed in house using our high-performance computer, ‘Hydra’. We use a pipeline that accepts RNA-seq data as input and produces tables of predictions for differentially expressed genes. Read alignment is performed using Tophat^65^. We use HTSeq^66^ to generate read counts, using the ENSEMBL GRCh37 annotations for reference. In order to determine those genes that are differentially expressed (DE), our pipeline makes use of three methods from the R Bioconductor package: DESeq^67^, edgeR^68^ and baySeq^69^. DESeq and edgeR are both competitive with the state of the art, while baySeq yields high precision in cases where there is no expression evident in one sample but weak or moderate expression in another sample^70^. The DE predictions from DESeq and edgeR allow us to establish high-confidence predictions that have low *p*-values from both methods. Data was organised using a spreadsheet and is filtered according to the edgeR adjusted *p*-value with a 0.05 cut-off applied. A total of 2,276 genes were identified as differentially expressed genes between the dexamethasone group and the control group taking into account the paired design across the 3 cell lines, and used for further analysis. For gene clustering and visualization, the gene expression matrix was analyzed by the supraHex package^71^. Genes with similar expression changes were self-organised onto nearby regions of a supra-hexagonal map. The resulting map was visualized to display sample-specific expression changes, and was also further partitioned to obtain 7 gene meta-clusters. For each meta-cluster, enrichment analysis was conducted using the function *dEnricher* in the dnet package^72^ to identify enriched functions (represented as Gene Ontology terms), enriched pathways (from BioCarta, KEGG and Reactome), and enriched transcription factor binding sites (TFBS).

### Comparisons with publicly available transcriptome data of MCN patients

Comparisons of differentially expressed genes against the MCN patient transcriptome data were carried out using the gene set enrichment analysis (GSEA) algorithm. In brief, MCN-specific gene signatures were first defined based on the MCN patient transcriptome data (obtained from the Nephroseq database ‘Ju Podocyte’; https://www.nephroseq.org). Differentially expressed genes were pre-ordered from the highest upregulations to the highest downregulations. Then, the function *dGSEA* in the dnet package was applied to determine the tendency of genes in a MCN-specific gene signature to be at the top or bottom of a pre-ordered differentially expressed genes. This tendency was quantified by running enrichment score, and the enrichment significance by p-value.

### Identification of the gene network in dexamethasone-treated podocytes

The dnet package was used to identify the gene network that contains groups of interconnected genes. Genes from the identified network are mostly significantly regulated in dexamethasone-treated podocytes but also contain a few less significant genes as linkers. Specifically, the *dNetPipeline* function in the package took as inputs a list of differentially expressed genes with the significance level (e.g. FDR), projected these genes onto the human protein association data from the STRING database (highest confidence > 900)^73^, and output a 56-gene network (under the tolerable thresholds of FDR < 1E-4). For a better visualization of the gene network structure, communities in the network were also detected via a span-glass model and simulated annealing.

### Real-time reverse transcription (RT)-PCR analysis

RNA extracted from cells using TRIzol (Invitrogen) method according to manufacturer’s instructions. High capacity RNA to cDNA kit (Applied Biosystems) was used to make cDNA as per the manufacturer’s instructions. Real-time PCR using SYBR Green (Sigma) was performed using StepOnePlus Real-Time PCR System (Applied Biosystem) and StepOne Software v2.1. Data was analyzed using ΔΔ Ct method. Primer sequences used are listed below:

GAPDH forward primer 5′-TGATGACATCAAGAAGGTGG-3′, reverse primer 5′-TTTCTTACTCCTTGGAGGCC-3′;

GILZ forward primer 5′-GATGTGGTTTCCGTTAAGC-3′, reverse primer 5′- CTCTCTCACAGCATACATCAG-3′;

GADD45B forward primer 5′-ATGAATGTGGACCCAGACAGC-3′, reverse primer 5′-GCGTGAAGTGGATTTGCAGG-3′;

TMEM2 forward primer 5′-GCACGGGGTTACTGTTTTTGT-3′, reverse primer 5′-GAGTCTGTGGCTGCTTGGAT-3′;

GLUL forward primer 5′-CCAAGTGTGTGGAAGAGTTGC-3′, reverse primer 5′-GCAGCAGGCACGAGATACAT-3′;

NEDD9 forward primer 5′-GGGACCTTCTCGCTTTCATCT-3′, reverse primer 5′-GTGGGTTGAGCCGTTTTCCT-3′;

ZEB1 forward primer 5′-TCATTGTGGAGAGATGACTTGT-3′, reverse primer 5′-AGCGGCAACAGCTCAATATG-3′;

ADD1 forward primer 5′-GGGGCCTAAAAGCAAGGTTC-3′, reverse primer 5′-GGATCTCACAGGCAACCACA-3′.

Four independent experiments were carried out on separate passages of cells. Two-tailed Student’s unpaired t-test was used to identify statistical significance. P values of <0.05 were taken to indicate statistical significance.

### Data availability

RNA-seq data in this study has been deposited in NCBI’s Gene Expression Omnibus and is accessible through GEO Series accession number GSE80651.

## Additional Information

**How to cite this article**: Jiang, L. *et al*. RNA sequencing analysis of human podocytes reveals glucocorticoid regulated gene networks targeting non-immune pathways. *Sci. Rep.* **6**, 35671; doi: 10.1038/srep35671 (2016).

## Supplementary Material

Supplementary Figure 1

Supplementary Table 1

Supplementary Table 2

Supplementary Table 3

Supplementary Table 4

## Figures and Tables

**Table 1:** Actin/microtubule-related genes upregulated by dexamethasone.

Cluster	Molecular Function	P-value	FDR	Gene Name
5	actin binding	0.0011	0.015	KLHL4, SHROOM2, DIAPH2, FSCN1, SNTA1, TAGLN, TPM2, HOMER2, DSTN,DIXDC1, FGD4, GAS2L3
6	actin binding	2.5E-12	9.60E-11	MSN, MYO1C, MYO9A, CALD1, CAPG,CFL1, CNN2, DIAPH1, EMD, FLNB, ABLIM1, OPHN1, PLEC, PLS3, TLN1, TPM4
				VASP, VCL, WIPF1, WDR1, ARPC1B, ACTR2, CAP1, PDLIM5, GIPC1, PALLD, SPTBN5, SSH3, PARVA, MTSS1L, SYNPO2
6	structural constituent of cytoskeleton	0.00002	0.00025	MSN, TUBA1B, TUBB4B, TLN1, ARPC1B, TUBB, TUBB2B, ACTG1, NEFL, SORBS3
7	microtubule binding	0.000031	0.00035	MAST2, CENPE, KIF23, KIF13B, KIFC2, KIF24, RACGAP1, MAP4, MAP6D1, PRC1, SBDS, KATNAL1
7	actin binding	0.001	0.0049	DAG1, TPM3, ADD1, CFL2, EPS8, UTRN, YWHAH, ARPC5, EPB41L3, RUSC1, TMOD3, ANLN,INF2, CLMN, DIAPH3

**Table 2:** List of genes in network communities upregulated by dexamethasone.

Name	Description	Log2(FC)	P-value	FDR
*C1*:RAS/MAPK signalling				
DUSP1	dual specificity phosphatase 1	0.89	2.9E-31	7.3E-29
TSC22D3	TSC22 domain family, member 3	1.57	4.7E-31	1.1E-28
CCND3	cyclin D3	0.87	2.0E-26	3.7E-24
EFNB2	ephrin-B2	0.78	7.8E-23	1.1E-20
SGK1	serum/glucocorticoid regulated kinase 1	1.15	1.9E-22	2.5E-20
ZFP36	ZFP36 ring finger protein	0.88	6.1E-19	6.2E-17
KRT8	keratin 8	0.91	1.8E-12	9.3E-11
SMYD2	SET and MYND domain containing 2	0.42	1.9E-10	7.4E-09
ITSN1	intersectin 1 (SH3 domain protein)	0.37	1.1E-08	3.3E-07
CAPN2	calpain 2, (m/II) large subunit	0.49	1.5E-06	3.0E-05
RRAS	related RAS viral (r-ras) oncogene homolog	0.32	4.7E-06	8.4E-05
RB1	retinoblastoma 1	0.24	4.9E-04	4.9E-03
RAF1	v-raf-1 murine leukemia viral oncogene homolog 1	0.19	3.3E-03	2.4E-02
MAPK1	mitogen-activated protein kinase 1	0.20	4.1E-03	2.9E-02
*C2*:Actin cytoskeleton regulation				
TGFBR1	transforming growth factor, beta receptor 1	1.30	6.3E-42	2.9E-39
CHRD	chordin	1.18	3.6E-37	1.3E-34
SERPINE1	serpin peptidase inhibitor, clade E (nexin, plasminogen activator inhibitor type 1), member 1	1.58	6.7E-35	2.2E-32
PER1	period circadian clock 1	1.52	9.7E-34	2.7E-31
CTTNBP2	cortactin binding protein 2	0.73	4.0E-14	2.5E-12
CHRDL1	chordin-like 1	0.55	1.7E-11	7.8E-10
TGFB2	transforming growth factor, beta 2	0.47	1.4E-09	4.7E-08
NPAS2	neuronal PAS domain protein 2	0.56	6.4E-09	2.0E-07
VASP	vasodilator-stimulated phosphoprotein	0.40	3.9E-08	1.0E-06
TGFBR2	transforming growth factor, beta receptor II (70/80kDa)	0.38	4.8E-08	1.3E-06
LPP	LIM domain containing preferred translocation partner in lipoma	0.37	1.6E-07	3.8E-06
MYL9	myosin, light chain 9, regulatory	0.57	1.9E-07	4.4E-06
PPP1CB	protein phosphatase 1, catalytic subunit, beta isozyme	0.36	3.3E-07	7.5E-06
ACTN1	actinin, alpha 1	0.54	8.2E-07	1.7E-05
PPP2CB	protein phosphatase 2, catalytic subunit, beta isozyme	0.34	2.7E-06	5.1E-05
BAMBI	BMP and activin membrane-bound inhibitor	0.41	2.7E-06	5.1E-05
*C3*:Integrin binding and extracellular matrix organization				
DCN	decorin	1.59	2.4E-65	3.6E-62
IRS2	insulin receptor substrate 2	1.51	4.0E-52	3.0E-49
COL4A3	collagen, type IV, alpha 3 (Goodpasture antigen)	1.09	1.5E-18	1.5E-16
PTPN1	protein tyrosine phosphatase, non-receptor type 1	0.63	1.1E-15	7.9E-14
COL4A4	collagen, type IV, alpha 4	0.89	4.4E-14	2.7E-12
TERT	telomerase reverse transcriptase	0.75	3.2E-09	1.0E-07
LUM	lumican	0.48	4.9E-09	1.5E-07
COL8A1	collagen, type VIII, alpha 1	0.69	2.7E-08	7.4E-07
PTPRJ	protein tyrosine phosphatase, receptor type, J	0.43	2.9E-08	7.9E-07
PDGFB	platelet-derived growth factor beta polypeptide	0.37	1.7E-07	4.1E-06
EPS8	epidermal growth factor receptor pathway substrate 8	0.37	5.8E-07	1.3E-05
RUVBL2	RuvB-like AAA ATPase 2	0.31	1.4E-06	2.7E-05
BTC	betacellulin	0.53	5.7E-06	9.9E-05
COL5A1	collagen, type V, alpha 1	0.33	1.3E-04	1.5E-03
SDC3	syndecan 3	0.28	1.9E-04	2.2E-03
EGFR	epidermal growth factor receptor	0.23	3.6E-03	2.6E-02
*C4*:Calcium signalling				
SNTA1	syntrophin, alpha 1	0.67	3.0E-15	2.0E-13
DGKI	diacylglycerol kinase, iota	0.55	3.6E-14	2.2E-12
EDN1	endothelin 1	0.67	2.4E-13	1.4E-11
S100A10	S100 calcium binding protein A10	0.58	1.2E-11	5.8E-10
ITPK1	inositol-tetrakisphosphate 1-kinase	0.41	8.5E-10	3.0E-08
ITPKA	inositol-trisphosphate 3-kinase A	0.81	8.0E-09	2.4E-07
EDN2	endothelin 2	1.01	1.2E-08	3.4E-07
ANXA2	annexin A2	0.46	8.2E-08	2.1E-06
CALM1	calmodulin 1 (phosphorylase kinase, delta)	0.38	6.8E-07	1.4E-05
PLCB3	phospholipase C, beta 3 (phosphatidylinositol-specific)	0.31	1.1E-06	2.2E-05
