# Supplementary material for: RNA sequencing analysis of human podocytes reveals glucocorticoid regulated gene networks targeting non-immune pathways
Source: Sci Rep. 2016 Oct 24;6:35671. doi: 10.1038/srep35671 (PMC5075905; doi:10.1038/srep35671)
Supplement: Supplementary Figure 1 [file srep35671-s1.pdf]

RNA sequencing analysis of human podocytes reveals glucocorticoid regulated gene networks targeting non-immune pathways

Lulu Jiang[1], Charles CT Hindmarch[2], Mark Rogers[3], Colin Campbell[3], Christy Waterfall[4], Jane Coghill[4], Peter W Mathieson[5] & Gavin I Welsh[1]

Address for correspondence: Dr Gavin I Welsh, Bristol Renal, School of Clinical Sciences, University of Bristol, Dorothy Hodgkin Building, Whitson Street, Bristol, BS1 3NY, UK. Tel: +44 117 331 3086. e-mail: g.i.welsh@bristol.ac.uk

[1] Bristol Renal, School of Clinical Sciences, University of Bristol, Bristol, UK
[2] Department of Biomedical and Molecular Sciences, Queen's University, Kingston, ON, Canada and Department of Physiology, Faculty of Medicine, University of Malaya, Kuala Lumpur, Malaysia
[3]Department of Engineering Mathematics, University of Bristol, Bristol, UK
[4] Bristol Genomics Facility, School of Biological Sciences, University of Bristol, Bristol, UK
[5] President's Office, The University of Hong Kong, Hong Kong, China

Conflict of interest: The authors have declared that no conflict of interest exists

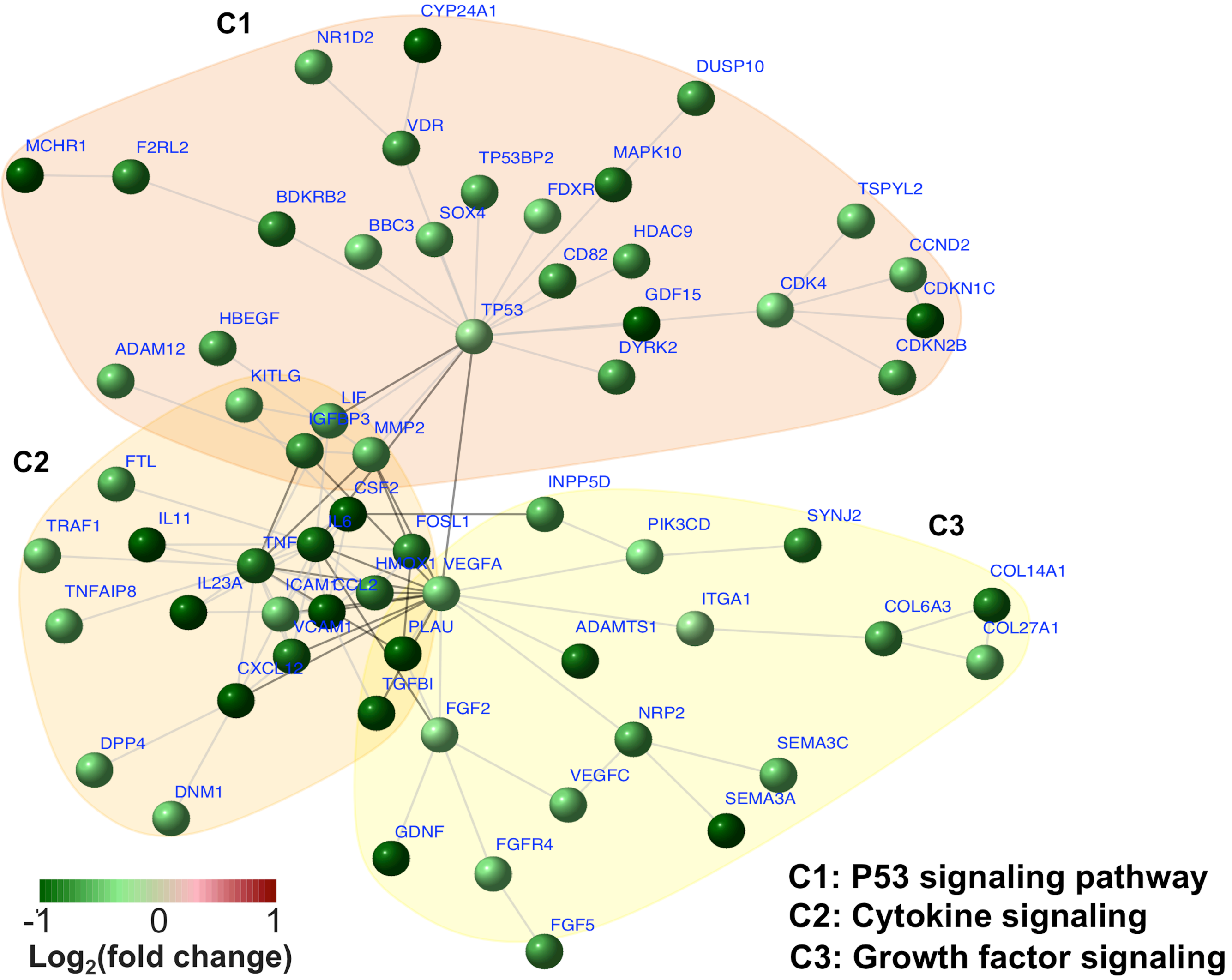

C1
C2
C3
CYP24A1
NR1D2
DUSP10
VDR
MCHR1
F2RL2
TP53BP2
MAPK10
BDKRB2
FDXR
TSPYL2
SOX4
BBC3
HDAC9
CCND2
CD82
CDK4
CDKN1C
GDF15
TP53
HBEGF
DYRK2
CDKN2B
ADAM12
KITLG
LIF
IGFBP3
MMP2
FTL
CSF2
INPP5D
IL6
FOSL1
TRAF1
IL11
PIK3CD
SYNJ2
TNF
HMOX1
VEGFA
COL14A1
IL23A
ICAM1
CCL2
TNFAIP8
ITGA1
COL6A3
VCAM1
PLAU
ADAMTS1
COL27A1
CXCL12
TGFBI
FGF2
NRP2
SEMA3C
DPP4
VEGFC
SEMA3A
DNM1
GDNF
FGFR4
FGF5
-1
0
1
Log2(fold change)
C1: P53 signaling pathway
C2: Cytokine signaling
C3: Growth factor signaling
